# Anthropometric indices for non-pregnant women of childbearing age differ widely among four low-middle income populations

**DOI:** 10.1186/s12889-017-4509-z

**Published:** 2017-07-24

**Authors:** K Michael Hambidge, Nancy F Krebs, Ana Garcés, Jamie E Westcott, Lester Figueroa, Shivaprasad S Goudar, Sangappa Dhaded, Omrana Pasha, Sumera Aziz Ali, Antoinette Tshefu, Adrien Lokangaka, Vanessa R Thorsten, Abhik Das, Kristen Stolka, Elizabeth M McClure, Rebecca L Lander, Carl L Bose, Richard J Derman, Robert L Goldenberg, Melissa Bauserman

**Affiliations:** 1 University of Colorado Denver, Aurora, CO USA; 2 INCAP (Instituto de Nutrición de Centro América y Panamá), Guatemala City, Guatemala; 3 KLE University’s Jawaharlal Nehru Medical College, Belagavi, Karnataka India; 4 Aga Khan University, Karachi, Pakistan; 5 Kinshasa School of Public Health, Kinshasa, Democratic Republic of the Congo; 6 RTI International, Research Triangle Park, NC USA; 7 University of North Carolina, Chapel Hill, NC USA; 8 Thomas Jefferson University, Philadelphia, PA USA; 9 Columbia University, New York, NY USA

**Keywords:** Height, Weight, Stunting, Underweight, Overweight/obesity, Body mass index, Mid-upper arm circumference, Waist-hip ratio, Non-pregnant women, Low middle income countries, Rural, Multi-site, Democratic Republic of the Congo, Guatemala, India, Pakistan

## Abstract

**Background:**

Maternal stature and body mass indices (BMI) of non-pregnant women (NPW) of child bearing age are relevant to maternal and offspring health. The objective was to compare anthropometric indices of NPW in four rural communities in low- to low-middle income countries (LMIC).

**Methods:**

Anthropometry and maternal characteristics/household wealth questionnaires were obtained for NPW enrolled in the Women First Preconception Maternal Nutrition Trial. Body mass index (BMI, kg/m^2^) was calculated. Z-scores were determined using WHO reference data.

**Results:**

A total of 7268 NPW participated in Equateur, DRC (*n* = 1741); Chimaltenango, Guatemala (*n* = 1695); North Karnataka, India (*n* = 1823); and Thatta, Sindh, Pakistan (*n* = 2009). Mean age was 23 y and mean parity 1.5. Median (P25-P75) height (cm) ranged from 145.5 (142.2–148.9) in Guatemala to 156.0 (152.0–160.0) in DRC. Median weight (kg) ranged from 44.7 (39.9–50.3) in India to 52.7 (46.9–59.8) in Guatemala. Median BMI ranged from 19.4 (17.6–21.9) in India to 24.9 (22.3–28.0) in Guatemala. Percent stunted (<−2SD height for age z-score) ranged from 13.9% in DRC to 80.5% in Guatemala; % underweight (BMI <18.5) ranged from 1.2% in Guatemala to 37.1% in India; % overweight/obese (OW, BMI ≥25.0) ranged from 5.7% in DRC to 49.3% in Guatemala. For all sites, indicators for higher SES and higher age were associated with BMI. Lower SES women were underweight more frequently and higher SES women were OW more frequently at all sites. Younger women tended to be underweight, while older women tended to be OW.

**Conclusions:**

Anthropometric data for NPW varied widely among low-income rural populations in four countries located on three different continents. Global comparisons of anthropometric measurements across sites using standard reference data serve to highlight major differences among populations of low-income rural NPW and assist in evaluating the rationale for and the design of optimal intervention trials.

**Trial registration:**

ClinicalTrials.gov #NCT01883193 (18 June 2013, retrospectively registered)

## Background

The height, weight, and body composition of women prior to conception have important implications for the subsequent health of the mother during pregnancy, delivery, and postpartum and for the development of her offspring both pre-and post-natally [1–7].

Information on anthropometry of non-pregnant women (NPW) is especially important in low and low-middle income countries (LMIC) where millions of women of childbearing age have anthropometric evidence of an adverse environment, including recent or/and long term undernutrition and where the rate of increase in overweight/obesity (OW) may now exceed that in more affluent countries [4]. National Health and Nutrition Surveys [8–14] and subsequent reports based on these data [3, 5, 8, 15–17] have provided most of the available anthropometric data. Though these reports have included regional data within countries, this is variable. Three-quarters of the variation in under-5 y of age mortality in sub-Saharan Africa has been estimated to be attributable to factors that vary within countries, in contrast to the one quarter that vary between countries [18]. Both linear growth and BMI of rural populations in LMIC can differ from those of corresponding urban populations [19]. These considerations highlight the potential value of data for selected communities within countries, including attention to rural populations.

To augment current knowledge, we report anthropometric data describing the cohort of women enrolled in the Women First preconception maternal nutrition trial [20], all of whom were of reproductive age and anticipated becoming pregnant. The primary goal of the parent study is to determine the effects of maternal nutrition supplements commenced prior to conception in rural sites in South Asia, sub-Saharan Africa, and Central America on offspring growth. This paper includes data on all consented NPW who had anthropometry and completed questionnaires prior to any intervention. These anthropometric data collected uniformly across four diverse sites, each in rural LMIC settings spanning three continents, served to provide a perspective on both the anthropometric heterogeneity as well as similarities in populations of women whose offspring’s birth anthropometry is the primary outcome of the trial.

## Objectives

The primary objectives of this paper were: 1) To characterize the maternal height distributions for the four sites and compare prevalence of stunting by site; 2) To characterize the maternal weight, BMI, and other anthropometric distributions for the four sites; 3) To compare prevalence of underweight (UW) and OW by site. A secondary objective was to identify associations between selected maternal and environmental characteristics with maternal height and BMI (UW, normal weight (NW), and OW).

## Methods

### Study design

This was a prospective observational study undertaken before any intervention in all women enrolled in the parent Women First study during 2013–14 [20] who had anthropometry and completed questionnaires on maternal characteristics and household wealth. The rural sites that provide cohorts for this study are located in Equateur Province, Democratic Republic of the Congo (DRC); Department of Chimaltenango, Western Highlands of Guatemala; Belagavi, North Karnataka, India; and Thatta, Sindh Province, Pakistan.

### Subjects

Among the inclusion criteria for participants in the Women First trial were: not currently pregnant and with intent/expectation to conceive during the following 18 mo, age 16–35 y, and planning to stay in the study area. Additionally, for women who had had a previous pregnancy with duration more than 20 wk, the delivery or termination had not occurred within the previous two mo. Women who were using or planning to use contraceptives, those who had allergies to groundnuts, and those with a hemoglobin measurement ≤8 g/dL were excluded.

Women who had recently completed a previous pregnancy were identified in part through an ongoing prospective pregnancy registry at all sites, the Maternal & Neonatal Health Registry of the *Eunice Kennedy Shriver* NICHD Global Network for Women’s and Children’s Health Research (Global Network) [21]. Others, including nulliparous women, were recruited through household surveys, local health centers, word-of-mouth, and local advertising. Data from all women who were enrolled and who had anthropometry were included. Less than 15% of parous participants were enrolled before 6 mo postpartum, except in the Pakistan site where >99% of the parous participants were less than 6 mo postpartum. The earlier post-partum recruitment at this site reflected concern about short inter-pregnancy intervals.

This project was approved by the Colorado Multiple Institutional Review Board in Colorado, the national ethics committees for each of the four sites, the data coordinating center (DCC) at RTI International (Research Triangle Park, NC, USA), and the Data Monitoring Committee of the Global Network prior to implementation. The study is registered in ClinicalTrials.gov (NCT01883193, initial release 18 June 2013). Written informed consent was obtained from the human subjects prior to participation.

### Anthropometry

Maternal height, weight, head circumference, mid-upper arm circumference (MUAC), waist, and hip circumference measurements were obtained by a specially trained mobile assessment team at each site utilizing standardized calibrated study equipment at the local health centers or in the participant home. Subjects were lightly clothed with no shoes. Height was recorded to the nearest 0.1 cm and weight to the nearest 0.1 kg. BMI (kg/m^2^) was calculated from recorded height and weight.

### Questionnaires

Demographic, obstetric history, and socio-economic status (SES) data were obtained in the home by the trained Home Visitor Research Assistants using baseline questionnaires. The questionnaires were reviewed by the centrally trained supervising staff for completeness, legibility, and accuracy prior to data entry.

### Data management

After review, the local data management centers keyed the data into password protected servers and securely transmitted the data to the DCC. During data entry, consistency and range checks were carried out through the data management system. Across-form and additional consistency edits were completed at the DCC and resolved locally.

### Outcome variables

1) Height (cm): mean, standard deviation (SD), median (range); 2) percentage of women who were stunted (<−2 SD for height for age z-scores (HAZ)) and severely stunted (<−3 SD HAZ) [22] using WHO standards for females, with stunting and severe stunting not exclusive [23]; 3) percentage of women who were UW (BMI <18.5), NW (BMI = 18.5 < 25.0), OW (BMI ≥25); 4) head circumference (cm): mean (SD); median (range); 5) MUAC: mean (SD); median (range); 6) percentage of women with MUAC <23 cm; 7) waist-hip ratio (WHR): mean (SD); median (range); 8) percentage of women whose WHR was low (< 0.80), moderate (0.80 – ≤0.85), or high risk (> 0.85) for metabolic disease [24, 25].

### Covariates

Covariates of primary interest, including commonly reported indicators of higher SES [26, 27], were tallied. These indicators were: 1) electricity; 2) improved water source (i.e. faucet inside house, public tap, other pipe source, public well, mechanical pump well, bore well within home, protected water source); 3) sanitation (own flush toilet); 4) man made flooring; 5) improved cooking fuels; and 6) household assets (more than one of radio, television, telephone, bike, motor bike/motor scooter, refrigerator or household owns a car or truck). The proportions of families with 0, 1–2, 3–4, or 5–6 of these indicators present were calculated.

### Analysis approach

This paper provides a cross-sectional analysis that describes baseline characteristics for participants in the Women First trial [20]. We hypothesized that maternal stunting, BMI, and other measures of body composition would vary by site and that specific participant characteristics are associated with height and BMI. The paper focuses on comparisons of maternal anthropometric measurements across sites. Also included is an assessment of associations within sites between selected maternal characteristics and BMI. The distributions of continuous measures for height and BMI were calculated by site. Frequencies and percentages were calculated for categorical anthropometric measures including height, BMI, MUAC, and WHR by site. Ninety-five percent Wilson confidence limits for the binomial proportions were calculated [28]. Differences between stunting, severe stunting, and BMI by site were assessed using chi-square tests and ANOVA analysis. Chi-square tests were used to assess associations between BMI and maternal characteristics. Pearson correlation coefficients were used to examine correlations between both MUAC and WHR and BMI for individual and combined sites. All *p*-values provided for characteristics are for descriptive purposes only and do not control for multiple tests. All analyses were done by the DCC at RTI using SAS version 9.4 (SAS Institute, Cary, NC, USA).

## Results

Of the 7387 women who consented to participate in the Women First trial, 7268 (98%) had height, weight, head circumference, MUAC, hip and waist circumference measurements taken and completed the questionnaires. Selected maternal characteristics by site, including age, parity, breastfeeding history, education, and SES, are given in Table 1. The women ranged in age from 15 to 37 y (mean age 23.4 y). A modest percentage of participants were <19 y of age (15% overall). Half of the participants in Guatemala and Pakistan were 25 y of age or more. Parity ranged from 0 to 6 (mean parity 1.5). Approximately one-third of participants were nulliparous at three sites but only a small number in Guatemala (8%). Less than one third was para three or more with a very low percentage in this category for India (6%). A high percentage of Pakistani women had no formal education (82%). At the other extreme, a high percentage of women in India had secondary education or beyond (77%). Comparison of SES between sites indicated higher SES in Guatemala and India, where nearly 90% of women had three or more of the indicators present. In contrast, in DRC, 98% of women had two or fewer indicators present. Women in Pakistan had slightly higher tallies than DRC with 80% of women having 1–4 indicators present. The most common indicator across sites was access to improved water, ranging from 39% in DRC to 99% in India. Use of improved cooking fuel was the least common indicator across sites. Overall, less than 15% of all women reported cooking with electricity, LPG, natural gas, kerosene, or coal.

**Table 1:** Selected characteristics of non-pregnant women of childbearing age by site

Characteristic	Equateur Province, DRC	Chimaltenango, Guatemala	N Karnataka, India	Thatta, Pakistan
Maternal age, Mean (SD)	22.9 (4.9)	24.8 (4.5)	22.2 (3.6)	23.6 (4.2)
Age categories, n (%)	1741	1695	1823	2009
15–18 y	398 (22.9)	136 (8.0)	276 (15.1)	275 (13.7)
19–24 y	711 (40.8)	711 (41.9)	1101 (60.4)	791 (39.4)
25–37 y	632 (36.3)	848 (50.0)	446 (24.5)	943 (46.9)
Parity, Mean (SD)	1.6 (1.4)	2.0 (1.2)	1.0 (0.9)	1.6 (1.6)
Parity categories, n (%)	1741	1695	1823	2009
0	513 (29.5)	135 (8.0)	602 (33.0)	759 (37.8)
1–2	772 (44.3)	1068 (63.0)	1116 (61.2)	655 (32.6)
≥ 3	456 (26.2)	492 (29.0)	105 (5.8)	595 (29.6)
Still breastfeeding baby among those who breastfed their last baby, n/N (%)	848/1179 (71.9)	1021/1500 (68.1)	869/1154 (75.3)	1053/1098 (95.9)
Maternal education (y), Mean (SD)	4.1 (3.1)	5.1 (3.3)	8.3 (3.7)	1.2 (2.8)
Maternal education categorized, n (%)	1741	1695	1823	2009
No formal schooling	371 (21.3)	137 (8.1)	139 (7.6)	1640 (81.6)
Primary	1001 (57.5)	1148 (67.7)	275 (15.1)	235 (11.7)
Secondary +	369 (21.2)	410 (24.2)	1409 (77.3)	134 (6.7)
Indicators of higher SES^a^, n (%)				
Household has electricity	10 (0.6)	1587 (93.6)	1698 (93.1)	1280 (63.7)
Household has access to improved water source	683 (39.2)	1512 (89.2)	1808 (99.2)	1732 (86.2)
Household has own flush toilet	10 (0.6)	770 (45.4)	383 (21.0)	667 (33.2)
Flooring of dwelling is man made	49 (2.8)	1274 (75.2)	840 (46.1)	869 (43.3)
Household uses improved cooking fuel	3 (0.2)	214 (12.6)	506 (27.8)	244 (12.1)
Household has assets	377 (21.7)	1245 (73.5)	1610 (88.3)	876 (43.6)
Tally of indicators of higher SES^a^, n (%)	1741	1695	1823	2009
0 indicators present	884 (50.8)	2 (0.1)	0 (0.0)	56 (2.8)
1–2 present	829 (47.6)	192 (11.3)	184 (10.1)	894 (44.5)
3–4 present	28 (1.6)	965 (56.9)	1177 (64.6)	721 (35.9)
5–6 present	0 (0.0)	536 (31.6)	462 (25.3)	338 (16.8)

^a^ In order to compare socio-economic status (SES) across sites, we looked at commonly reported indicators of SES, namely 1) electricity, 2) improved water source, 3) sanitation, 4) man-made flooring, 5) improved cooking fuels, and 6) household assets. Improved water source includes faucet inside house, public tap, other pipe source, public well, mechanical pump well, bore well within home, protected water source; improved cooking fuel includes electricity, LPG, natural gas, kerosene, or coal; assets include more than one of: radio, TV, telephone, bike, motorcycle/motor scooter, or refrigerator, or household owns a car or truck. We tallied these six indicators and reported the proportion of families without any, with 1–2 indicators, with 3–4 indicators, and with 5–6 indicators present

**Fig. 1:**
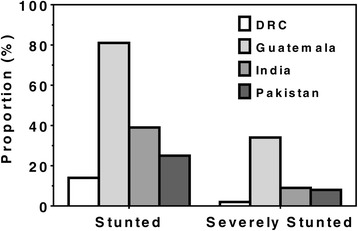
Women First trial: Prevalence of stunting and severe stunting in non-pregnant women of childbearing age by site. Stunted defined as -2SD height for age z-scores (HAZ). Severely stunted defined as -3SD HAZ [22]

The mean (SD) height ranged from a low of 145.6 (5.0) cm in Guatemala to a high of 156.1 (6.2) in DRC. Corresponding stunting prevalences (95% confidence intervals (CI)) were a high of 80.5% (78.5, 82.3) in Guatemala and a low of 13.9% (12.4, 15.6) in DRC. Stunting rates were also high in India and Pakistan (Table 2 **,** Fig. 1).

**Table 2:** Anthropometric indices of non-pregnant women of childbearing age by site

Characteristic	Equateur Province, DRC	Chimaltenango, Guatemala	N Karnataka, India	Thatta, Pakistan	*p*-value^1^
Weight (kg), n	1741	1695	1823	2008	
Mean (SD)	50.7 (7.6)	54.2 (10.0)	46.1 (8.6)	46.2 (7.5)	
Median (P25, P75)	50.0 (46.0, 55.0)	52.7 (46.9, 59.8)	44.7 (39.9, 50.3)	45.0 (41.0, 49.5)	
Min - Max	31.0–105.0	32.0–101.9	28.7–90.7	30.0–94.5	
Height (cm), n	1741	1695	1823	2009	
Mean (SD)	156.1 (6.2)	145.6 (5.0)	151.3 (5.7)	152.4 (6.2)	
Median (P25, P75)	156.0 (152.0, 160.0)	145.5 (142.2, 148.9)	151.3 (147.7, 155.2)	152.0 (149.5, 156.3)	
Min - Max	134.5–180.2	127.0–163.4	133.8–173.4	130.6–178.0	
Stunting, n (%)^a^	242 (13.9)	1364 (80.5)	716 (39.3)	511 (25.4)	<0.0001
Severe stunting, n (%)	33 (1.9)	567 (33.5)	156 (8.6)	168 (8.4)	<0.0001
Body Mass Index (BMI; kg/m^2)^, n	1741	1695	1823	2008	
Mean (SD)	20.8 (2.6)	25.5 (4.3)	20.1 (3.5)	19.9 (3.0)	<0.0001
Median (P25, P75)	20.6 (19.1, 22.1)	24.9 (22.3, 28.0)	19.4 (17.6, 21.9)	19.5 (17.8, 21.5)	
Min - Max	14.7–39.0	16.5–42.7	13.9–37.6	12.9–38.0	
BMI categories, n (%)					
Underweight: BMI <18.5	264 (15.2)	21 (1.2)	677 (37.1)	704 (35.1)	<0.0001
Normal weight: BMI 18.5 - <25	1378 (79.1)	838 (49.4)	968 (53.1)	1182 (58.9)	
Overweight/Obesity: BMI ≥25.0	99 (5.7)	836 (49.3)	178 (9.8)	122 (6.1)	
Head Circumference (cm), n	1741	1695	1823	2009	
Mean (SD)	53.5 (1.7)	53.7 (1.5)	52.5 (1.5)	52.9 (1.8)	
Median (P25, P75)	53.5 (52.4, 54.5)	53.6 (52.7, 54.7)	52.5 (51.5, 53.5)	53.0 (51.8, 54.0)	
Min - Max	47.0–59.0	41.8–63.7	47.0–59.2	47.0–60.0	
MUAC (cm), n	1741	1695	1823	2009	
Mean (SD)	25.7 (2.3)	27.2 (3.2)	24.0 (3.1)	23.2 (2.7)	
Median (P25, P75)	25.5 (24.1, 27.0)	26.9 (25.0, 29.1)	23.5 (21.8, 25.6)	23.0 (21.5, 24.6)	
Min - Max	19.8–37.5	19.4–57.9	17.0–38.1	15.8–50.0	
< 23.0, n (%)	152 (8.7)	94 (5.5)	740 (40.6)	982 (48.9)	<0.0001
Waist circumference (cm), n	1741	1693	1822	2004	
Mean (SD)	74.4 (5.6)	76.2 (9.7)	65.2 (8.7)	66.4 (7.0)	
Median (P25, P75)	74.0 (71.0, 77.4)	75.4 (69.4, 82.1)	63.5 (59.0, 69.7)	65.2 (62.0, 70.0)	
Min - Max	56.3–112.0	44.1–114.5	47.2–108.0	44.4–101.0	
Hip circumference (cm), n	1740	1694	1823	2002	
Mean (SD)	87.1 (6.5)	93.7 (8.2)	85.0 (7.2)	84.7 (6.9)	
Median (P25, P75)	87.0 (83.0, 91.0)	92.7 (88.1, 98.3)	84.0 (80.0, 89.0)	84.0 (80.0, 88.5)	
Min - Max	50.0–124.0	45.8–132.6	56.0–118.3	47.0–123.6	
Waist-Hip Ratio (WHR), n	1740	1692	1822	1997	
Mean (SD)	0.86 (0.05)	0.81 (0.07)	0.77 (0.07)	0.78 (0.06)	<0.0001
Median (P25, P75)	0.86 (0.82, 0.89)	0.81 (0.77, 0.85)	0.76 (0.72, 0.80)	0.78 (0.75 0.82)	
Min - Max	0.61–1.50	0.44–1.86	0.57–1.24	0.55–1.21	
WHR Categorized, n (%)					
Low risk (≤0.80)	254 (14.6)	790 (46.7)	1398 (76.7)	1371 (68.7)	<0.0001
Moderate risk (>0.80- ≤ 0.85)	466 (26.8)	430 (25.4)	236 (13.0)	374 (18.7)	
High risk (>0.85)	1020 (58.6)	472 (27.9)	188 (10.3)	252 (12.6)	

^1^ *P*-values from chi-square tests and ANOVA analysis to assess for differences between stunting, severe stunting, BMI, MUAC <23.0, and waist-hip ratio WHR by site

^a^ Stunted defined as -2SD height for age z-scores (HAZ). This is 147.9 cm for 15 y, 148.9 cm for 16 y, 149.5 cm for 17 y, 149.8 cm for 18 y and 150 cm for 19+ y. Severely stunted defined as -3SD HAZ. This is 141.0 cm for 15 y, 142.2 cm for 16 y, 142.8 cm for 17 y, 143.2 cm for 18 y and 143.5 cm for 19+ y [23]

**Fig. 2:**
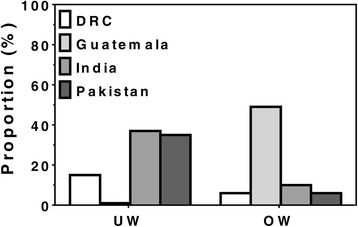
Women First trial: Prevalence of underweight (UW) and overweight/obesity (OW) in non-pregnant women of childbearing age determined by Body Mass Index (BMI) by site. UW defined as BMI <18.5; OW defined as BMI ≥25.0

Mean weight and BMI also varied substantially by site. The mean BMI ranged from a low of 19.9 kg/m^2^ in Pakistan to a high of 25.5 kg/m^2^ in Guatemala. More than one-third of women in the Indian and Pakistani sites had BMIs <18.5 kg/m^2^ compared with a modest prevalence of low BMIs in DRC and a prevalence approaching zero in Guatemala. In contrast, the BMI was ≥25 kg/m^2^ in almost 50% of women in Guatemala, indicative of OW with corresponding figures of <10% in the other sites (Table 2 **,** Fig. 2).

**Fig. 3:**
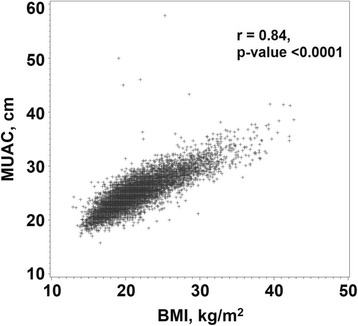
Women First trial: Correlation of mid-upper arm circumference (MUAC) with body mass index (BMI) for non-pregnant women of childbearing age from four rural low- low-middle- income populations. The correlation between MUAC and BMI while controlling for site was 0.84; *n* = 7267

MUAC correlated strongly with BMI with an overall Pearson partial correlation controlling for site of 0.84 (Fig. 3). The correlation was strongest for the women in India with a correlation of 0.92 and lowest in Pakistan (0.70) with intermediate correlation coefficients for DRC and Guatemala.

The mean (SD) waist circumference ranged from 65.2 (8.7) cm in India to 76.2 (9.7) in Guatemala (Table 2). The mean (SD) hip circumference ranged from 84.7 (6.9) cm in Pakistan to 93.7 (8.2) cm in Guatemala. The WHR was highest in DRC (mean = 0.9) where 58.6% (56.3, 60.9) of women had ‘high risk’ ratios of >0.85. ‘High risk’ prevalences (95% CI) were 27.9% (25.8, 30.1), 12.6% (11.2, 14.2), and 10.3% (9.0, 11.8) in Guatemala, Pakistan, and India respectively (Table 2). The overall partial correlation adjusted for site for WHR with BMI was 0.24 with a low of −0.05 in DRC and a high of 0.45 in India.

Maternal age and tally of indicators of higher SES within sites were associated with BMI at all sites (Table 3, *p* < 0.01). The results for SES are generally consistent across sites with a trend towards underweight women having lower SES, normal weight women having middle SES, and OW women having higher SES. Similarly, for all sites, younger women tended to be underweight, while older women tended to be OW. In DRC and Pakistan, maternal education was associated with BMI (*p* < 0.01), with OW women having higher education. There was no evident association between parity and BMI in DRC (*p* = 0.34), Guatemala (*p* = 0.84), or Pakistan (0.10). However, parity was associated with BMI in India (*p* = 0.007) with underweight women having lower parity. Maternal characteristics and household wealth had a very similar distribution for stunted and non-stunted women (Table 4).

**Table 3:** Characteristics of non-pregnant women of childbearing age by site and body mass index (BMI)^a^

	Equateur Province, DRC				Chimaltenango, Guatemala				N Karnataka, India				Thatta, Pakistan			
Characteristic	UW (*n* = 264)	NW (*n* = 1378)	OW (*n* = 99)	*p*-value^*^	UW (*n* = 21)	NW (*n* = 838)	OW (*n* = 836)	*p*-value^*^	UW (*n* = 677)	NW (*n* = 968)	OW (*n* = 178)	*p*-value^*^	UW (*n* = 704)	NW (*n* = 1182)	OW (*n* = 122)	*p*-value^*^
Maternal age categories, n (%)																
15–18 y	44 (16.7)	342 (24.8)	12 (12.1)	0.0026	3 (14.3)	91 (10.9)	42 (5.0)	<0.0001	121 (17.9)	134 (13.8)	21 (11.8)	<0.0001	121 (17.2)	145 (12.3)	8 (6.6)	<0.0001
19–24 y	112 (42.4)	556 (40.3)	43 (43.4)		13 (61.9)	402 (48.0)	296 (35.4)		438 (64.7)	579 (59.8)	84 (47.2)		307 (43.6)	450 (38.1)	34 (27.9)	
25–37 y	108 (40.9)	480 (34.8)	44 (44.4)		5 (23.8)	345 (41.2)	498 (59.6)		118 (17.4)	255 (26.3)	73 (41.0)		276 (39.2)	587 (49.7)	80 (65.6)	
Parity categories, n (%)																
0	64 (24.2)	420 (30.5)	29 (29.3)	0.33	2 (9.5)	65 (7.8)	68 (8.1)	0.84	240 (35.5)	306 (31.6)	56 (31.5)	0.0069	274 (38.9)	444 (37.6)	40 (32.8)	0.10
1–2	123 (46.6)	603 (43.8)	46 (46.5)		12 (57.1)	539 (64.3)	517 (61.8)		414 (61.2)	589 (60.8)	113 (63.5)		246 (34.9)	368 (31.1)	41 (33.6)	
≥ 3	77 (29.2)	355 (25.8)	24 (24.2)		7 (33.3)	234 (27.9)	251 (30.0)		23 (3.4)	73 (7.5)	9 (5.1)		184 (26.1)	370 (31.3)	41 (33.6)	
Maternal education categorized, n (%)																
No formal schooling	68 (25.8)	288 (20.9)	15 (15.2)	0.0001	1 (4.8)	70 (8.4)	66 (7.9)	0.65	49 (7.2)	82 (8.5)	8 (4.5)	0.29	589 (83.7)	975 (82.5)	75 (61.5)	<0.0001
Primary	153 (58.0)	802 (58.2)	46 (46.5)		12 (57.1)	567 (67.7)	569 (68.1)		101 (14.9)	151 (15.6)	23 (12.9)		77 (10.9)	134 (11.4)	24 (19.7)	
Secondary +	43 (16.3)	288 (20.9)	38 (38.4)		8 (38.1)	201 (24.0)	201 (24.0)		527 (77.8)	735 (75.9)	147 (82.6)		38 (5.4)	73 (6.2)	23 (18.9)	
Tally of indicators of higher SES^b^																
0 indicators present	162 (61.4)	690 (50.1)	32 (32.3)	<0.0001	0 (0.0)	2 (0.2)	0 (0.0)	0.0008	0 (0.0)	0 (0.0)	0 (0.0)	<0.0001	23 (3.3)	32 (2.7)	1 (0.8)	<0.0001
1–2 present	99 (37.5)	668 (48.5)	62 (62.6)		4 (19.0)	118 (14.1)	70 (8.4)		83 (12.3)	91 (9.4)	10 (5.6)		350 (49.7)	516 (43.6)	27 (22.1)	
3–4 present	3 (1.1)	20 (1.5)	5 (5.1)		14 (66.7)	478 (57.0)	473 (56.6)		458 (67.7)	623 (64.4)	96 (53.9)		238 (33.8)	434 (36.7)	49 (40.2)	
5–6 present	0 (0.0)	0 (0.0)	0 (0.0)		3 (14.3)	240 (28.6)	293 (35.0)		136 (20.1)	254 (26.2)	72 (40.4)		93 (13.2)	200 (16.9)	45 (36.9)	

^a^ Underweight (UW) defined as BMI <18.5; Normal weight (NW) as 18.5 – <25.0; Overweight/Obesity (OW) as ≥25.0

^b^ In order to compare socio-economic status (SES) across sites, we looked at commonly reported indicators of SES, namely 1) electricity, 2) improved water source, 3) sanitation, 4) man-made flooring, 5) improved cooking fuels, and 6) household assets. Improved water source includes faucet inside house, public tap, other pipe source, public well, mechanical pump well, bore well within home, protected water source; improved cooking fuel includes electricity, LPG, natural gas, kerosene, or coal; assets include: radio, TV, telephone, bike, motorcycle/motor scooter, or refrigerator, or household owns a car or truck. We tallied these six indicators and reported the proportion of families without any, with 1–2 indicators, with 3–4 indicators, and 5–6 of these indicators present

^*^ *P*-values from chi-square tests to assess for associations between BMI and characteristic of interest

**Table 4:** Characteristics of non-pregnant women of childbearing age by site and stunting

Characteristic	Equateur Province, DRC		Chimaltenango, Guatemala		N Karnataka, India		Thatta, Pakistan	
	Stunted (*n* = 242)	Not stunted (*n* = 1499)	Stunted (*n* = 1364)	Not stunted (*n* = 331)	Stunted (*n* = 716)	Not stunted (*n* = 1107)	Stunted (*n* = 511)	Not stunted (*n* = 1498)
Maternal age categories, n (%)								
15–18 y	57 (23.6)	341 (22.7)	105 (7.7)	31 (9.4)	96 (13.4)	180 (16.3)	88 (17.2)	187 (12.5)
19–24 y	107 (44.2)	604 (40.3)	572 (41.9)	139 (42.0)	429 (59.9)	672 (60.7)	197 (38.6)	594 (39.7)
25–37 y	78 (32.2)	554 (37.0)	687 (50.4)	161 (48.6)	191 (26.7)	255 (23.0)	226 (44.2)	717 (47.9)
Parity categories, n (%)								
0	84 (34.7)	429 (28.6)	95 (7.0)	40 (12.1)	238 (33.2)	364 (32.9)	212 (41.5)	547 (36.5)
1–2	104 (43.0)	668 (44.6)	851 (62.4)	217 (65.6)	436 (60.9)	680 (61.4)	157 (30.7)	498 (33.2)
≥ 3	54 (22.3)	402 (26.8)	418 (30.6)	74 (22.4)	42 (5.9)	63 (5.7)	142 (27.8)	453 (30.2)
Maternal education categorized, n (%)								
No formal schooling	81 (33.5)	290 (19.3)	122 (8.9)	15 (4.5)	67 (9.4)	72 (6.5)	420 (82.2)	1220 (81.4)
Primary	139 (57.4)	862 (57.5)	958 (70.2)	190 (57.4)	125 (17.5)	150 (13.6)	66 (12.9)	169 (11.3)
Secondary+	22 (9.1)	347 (23.1)	284 (20.8)	126 (38.1)	524 (73.2)	885 (79.9)	25 (4.9)	109 (7.3)
Tally of indicators of higher SES^a^ n (%)								
0 indicators present	165 (68.2)	719 (48.0)	2 (0.1)	0 (0.0)	0 (0.0)	0 (0.0)	10 (2.0)	46 (3.1)
1–2 present	75 (31.0)	754 (50.3)	175 (12.8)	17 (5.1)	91 (12.7)	93 (8.4)	244 (47.7)	650 (43.4)
3–4 present	2 (0.8)	26 (1.7)	771 (56.5)	194 (58.6)	479 (66.9)	698 (63.1)	174 (34.1)	547 (36.5)
5–6 present	0 (0.0)	0 (0.0)	416 (30.5)	120 (36.3)	146 (20.4)	316 (28.5)	83 (16.2)	255 (17.0)

^a^ In order to compare socio-economic status (SES) across sites, we looked at commonly reported indicators of SES, namely 1) electricity, 2) improved water source, 3) sanitation, 4) man-made flooring, 5) improved cooking fuels, and 6) household assets. Improved water source includes faucet inside house, public tap, other pipe source, public well, mechanical pump well, bore well within home, protected water source; improved cooking fuel includes electricity, LPG, natural gas, kerosene, or coal; assets include: radio, TV, telephone, bike, motorcycle/motor scooter, or refrigerator, or household owns a car or truck. We tallied these six indicators and reported the proportion of families without any, with 1–2 indicators, with 3–4 indicators, and 5–6 of these indicators present

## Discussion

The cohorts for each of the four Global Network sites participating in this study had several similarities that facilitated their inclusion in the Women First protocol. These similarities included their location in LMIC and, more specifically, in rural environments within these countries. Nevertheless, they were located in countries across three continents with substantial differences in culture, race, dietary intakes [unpublished data], demographic features, disease patterns, and socio-economic indicators. Interpretation of these anthropometric data depends in part on whether they are representative of the populations in which the Women First participants are located. Each of the four sites have participated in the Maternal Neonatal Health Registry of the Global Network which records extensive data on >95% of women giving birth in these Network clusters [29]. During the three-year period 2014–16, heights and weights were measured and BMIs calculated for greater than 113,000 women in these four sites. The mean heights (SD) matched closely the mean heights reported here with figures of 157.2 (6.9), 147.0 (5.4), 152.3 (5.5), and 154.7 (5.6) for the sites in DRC, Guatemala, India, and Pakistan respectively (unpublished data). Weights were obtained at the time of enrollment in the Registry at the first antenatal care visit, which could occur at any time during gestation. Mean weights and BMIs, as expected, were close to but slightly higher than for Women First. These comparisons provide reassurance that the Women First preconception anthropometric data are representative of the populations in which they are resident. Fewer data are available on maternal characteristics and SES for the larger populations. Years of education and the low level of formal schooling are very similar to the Women First data. The Registry had similar parity for Guatemala and India but higher parity for DRC and Pakistan.

At a time when there is increasing appreciation of the benefits to be derived from global anthropometric standards [22, 30], there is also recent documentation of population differences in body composition [31–33]. Genetic changes affecting population heights in relatively long-term response to the environment have been documented [34]. In this study, as will be discussed below, the Equateur, DRC, data may suggest genetic differences in body composition. However, at a population level, there is a strong case for using global standards as evidenced by the anthropometric growth reference data of the World Health Organization used in evaluating the data reported here [23]. This case has perhaps already been strengthened by the growing appreciation of the role of potentially reversible epigenetic changes in response to the environment including intergenerational effects [2, 35, 36]. Comparison of anthropometric data across sites has served not only to highlight the differences but also to focus attention on the outstanding features of these anthropometric data in each of the four individual sites. A specific goal in this instance has been to provide baseline data for a preconception maternal nutrition intervention trial. However, these data have also increased awareness of differences in environmental loads between sites and could also, for example, provide an explanation for different risk profiles.

Despite estimates of a decline in the global prevalence of short stature, there remains a high prevalence in some regions including our study sites in South Asia and Central America [4]. Obstetric complications associated with maternal short stature include a higher incidence of pelvic deformities and associated complications; a lower possibility of delivering vaginally and higher incidence of instrumental deliveries [37]. Maternal height is inversely associated with the risk of dystocia [38–40]. The adverse associations of maternal stunting and offspring morbidity/mortality during infancy/early childhood are of even greater concern. Analysis of data from 109 Demographic Health Surveys in 54 countries conducted between 1991 and 2008 determined that for each 1 cm increase in maternal height, there was a decreased risk of offspring mortality between 0 and 5 y of age [3]. It has been estimated that close to 6.5 million/y small-for-gestational age and/or preterm births in LMIC may be attributed to factors that are associated with short maternal stature [6]. Short maternal height is associated with childhood stunting and anemia [3, 5], adult morbidity, and reduced human capital [2, 41]. A comprehensive examination of the relationship of maternal height to offspring length/height has been reported by the Consortium on Health Oriented Research in Transitional Societies group [41, 42]. This was based on data from 7630 mother-child pairs from 5 birth cohorts in Brazil [43, 44], Guatemala [45], India [46], the Philippines [47], and South Africa [48]. Mothers with short stature (height < 150.1 cm) were estimated to be more likely to have a child stunted at 2 years (prevalence ratio (95% CI): 3.20 (2.80–3.60)) and as an adult 4.74 (4.13–5.44). Pertinent to the current report, there was no heterogeneity by site [7]. In the current study, the cohort in the Western Highlands of Guatemala (altitude approximately 7000 ft) had an even lower mean maternal height than the recent National Survey for this population and a higher prevalence of stunting [49]. Our data are, however, in accord with previous reports for the indigenous population of Guatemala [2, 9–11, 50–52]. Short stature in this population is likely to result from a combination of genetic or, most likely, epigenetic factors and the maternal environment during mothers’ early pre- and post-natal growth [2]. In contrast, at the other three sites, the prevalence of maternal stunting was low in comparison with recent national data. In the Indian site the prevalence was lower than the prevalence reported in the COHORT study [7, 46] and was also lower than in the National Demographic and Health Survey 2006 [12]. In Pakistan, the 2011 National Nutrition Survey [53], revealed wide geographical differences at the district level in stunting, underweight, and wasting prevalence [8]. We found rates for stunting, underweight, and obesity in the Thatta District, Sindh Province, that are comparable to those obtained for this poor district in the 2011 National Nutrition Survey [8]. Pakistan has been notable for the very slow improvement in prevalence of stunting since 1985 [8] and BMI in DRC were very similar to that of an earlier study of ours in Equateur [54] [unpublished data]. A recent DHS report for DRC did not include the mean height for adult women in Equateur, but did report a prevalence of stunting of 2% [13]. This is even lower than the prevalence observed in this study (Table 1). However, the prevalence of stunting in this study was very low compared with WHO regional data for both Sub-Saharan Africa and South Asia [55]. The current prevalence of stunting in women of childbearing age for the sites in DRC and Pakistan contrasts with the higher prevalence for children aged 1–2 y recently measured in these sites [56]. Length at this age is considered a strong predictor of adult height and other adverse consequences in adulthood [57], but these differences in rates of stunting between toddlers and adults support the concept of potential catch-up growth during intervening years [58]. In the DRC, measurements of the young children and the adult women were undertaken in precisely the same site and same population with no discernible environmental changes over the past 5 years.

Underweight, indicative of recent and current primary or secondary undernutrition, remains a major concern, again with regional differences globally. Data from the five-country COHORT study highlight the importance of preconception maternal underweight to their offspring from age 2 y to adulthood [7, 46]. Data from the 3rd Indian National Family Health Survey in 2005–6 [12] indicated that maternal underweight, independent of short stature, was a predictor of early childhood stunting/underweight. Overall prevalence of underweight (BMI <18.5 kg/m^2^) in Asia and Africa has declined slowly since 1980 to a level of approximately 15% in 2008 [1], though the level in South Asia remained as high as 25% in 2014 [59]. In comparison, more than one-third of the Indian and Pakistani women were underweight in the current study. In the DRC site, the prevalence of maternal underweight was reasonably comparable to UNICEF regional estimate for Sub-Saharan Africa in 2008 [55] and close to the figure of 13.4% for Equateur in the recent DRC DHS report [13]. This prevalence was higher than that recently observed for children aged 1–2 y in the same population [56]. The prevalence of low BMIs at sites in India and Pakistan underlines the probable need for commencing improvement in the nutritional status of women of childbearing age prior to pregnancy in these rural and rural small town communities. In notable contrast to the other three sites, the prevalence of maternal underweight in the Western Highlands of Guatemala in this study was only 1%, which was likely explicable at least in part to the more transitional economic status of this population.

Maternal obesity is associated with an increase in the risk of both gestational diabetes [60] and preeclampsia [61]. During labor and delivery, maternal obesity is associated with an increased risk of maternal death, hemorrhage, and infection. There is also a higher risk of early offspring mortality, birth trauma, and infection [1]. In the current study, determination of maternal BMI was facilitated by the commencement of the Women First trial prior to conception, thus avoiding the inaccuracies resulting from estimations of BMI during pregnancy, or the expected, but variably higher, BMI in the early postpartum period. In the Guatemalan site, the prevalence of OW was 49% in contrast to rates of 5–10% at the other three sites. Maternal OW in Guatemala can be associated with childhood stunting, referred to as the double burden of malnutrition which has been reported to be more prevalent in poor and middle socio-economic groups than in more wealthy households [52]. However, it is questionable whether BMI ≥25 kg/m^2^ for women with short stature reflects the same body composition as for those with height within the normal range [62]. This special, and probably long-term, challenge does not, however, minimize its clinical importance [4]. Rather, there appears to be a synergistic effect of short stature and obesity on maternal complications of obesity [63]. Neither should it divert attention from the global dimensions of OW to which developing/transitional countries contribute an increasing burden [59]. The apparent disconnect between the low prevalence of underweight and the high prevalence of ‘at risk’ high WHR in the DRC site is the datum most suggestive of a genetic influence at the population level. Coupled with the relatively high mean heights and low levels of stunting, the participants in the DRC do not have an apparent nutrition-related explanation. This was unexpected in view of their high level of reported ‘food insecurity’ [unpublished data] and their relatively low SES scores. In this study, Guatemala and India had similar tallies of higher SES; however, the indicators used were quite limited and the timeframe is uncertain over which these cohorts have been moving towards a quasi-transitional status. The prevalence of OW in the Guatemalan site was highest in the oldest age range and was also associated with higher tallies of indicators of SES, but was not associated with parity or level of education. Overall, indicators of SES and maternal age were associated with BMI across sites, providing some insight into potential contributors to both under- and overweight. Absolute wealth has been reported to predict overweight status among women of comparable ages to those in this study in 360 populations across 36 developing countries with some variation according to world region [32].

In summary, anthropometric data for NPW of child bearing age varied widely by site. Prevalence of underweight in the women is notable in the Indian and Pakistani sites, while the prevalence of stunting and OW are both high in the Guatemalan site. The data for the DRC site are inconsistent with a typical pattern of either under- or overnutrition. Indicators of SES and maternal age were associated with BMI across sites. Though the communities included were primarily low-income, the prevalence of stunting was lower than recent national/regional data for India, but the prevalence of underweight was higher in both India and Pakistan.

## Conclusions

Anthropometric indices varied widely between rural sites in LMIC located in South Asia, Sub-Saharan Africa, and Central America. Some of the heterogeneity can be attributed to identified maternal characteristics and to household wealth, despite the limitations of the latter indicators and the overall limited range of wealth. The majority of the anthropometric data is consistent with either current or recent environmental factors including under- and overnutrition. Population differences in height may be attributable to long-term intergenerational environmental factors, including epigenetic changes. However, a genetic contribution is also plausible, most evidently in the body proportions of the women in Equateur. These and similar data would have been useful in the planning stage of the Women First preconception maternal nutrition trial and other trials involving women of childbearing age and their offspring. They should also prove pertinent to interpretation of the results of such trials.

## Abbreviations

BMI: Body mass index
CI: Confidence intervals
DCC: Data coordinating center
DRC: Democratic Republic of the Congo
HAZ: Height for age z-score
LMIC: Low and middle income countries
MUAC: Mid-upper arm circumference
NPW: Non-pregnant women
NW: Normal weight
OW: Overweight/obesity
SD: Standard deviation
SES: Socio-economic status
UW: Underweight
